# Screening of Fungi for Biological Control of a Triatomine Vector of Chagas Disease: Temperature and Trypanosome Infection as Factors

**DOI:** 10.1371/journal.pntd.0005128

**Published:** 2016-11-17

**Authors:** Aline R. M. Garcia, Adriana de Paula Rocha, Camila C. Moreira, Silma L. Rocha, Alessandra A. Guarneri, Simon L. Elliot

**Affiliations:** 1 Department of Entomology, Federal University of Viçosa, Campus Universitário, Viçosa, Minas Gerais, Brazil; 2 Centro de Pesquisas René Rachou, Avenida Augusto de Lima, Belo Horizonte, Minas Gerais, Brazil; Universidade Federal do Rio de Janeiro, BRAZIL

## Abstract

Entomopathogenic fungi have been investigated as an alternative tool for controlling various insects, including triatomine vectors of the protozoan *Trypanosoma cruzi*, the etiological agent of Chagas disease. Here we tested the pathogenicity and virulence of ten isolates of the fungi *Metarhizium* spp. and *Beauveria bassiana* against *Rhodnius prolixus* and found all of the isolates to be virulent. We used two isolates (URPE-11 *Metarhizium anisopliae* and ENT-1 *Beauveria bassiana*) for further screening based on their prolific sporulation *in vitro* (an important property of fungal biopesticides). We characterized their virulences in a dose-response experiment and then examined virulence across a range of temperatures (21, 23, 27 and 30°C). We found isolate ENT-1 to maintain higher levels of virulence over these temperatures than URPE-11. We therefore used *B*. *bassiana* ENT-1 in the final experiment in which we examined the survival of insects parasitized with *T*. *cruzi* and then infected with this fungus (once again over a range of temperatures). Contrary to our expectations, the survival of insects challenged with the pathogenic fungus was greater when they had previously been infected with the parasite *T*. *cruzi* than when they had not (independent of temperature). We discuss these results in terms of aspects of the biologies of the three organisms. In practical terms, we concluded that, while we have fungal isolates of potential interest for development as biopesticides against *R*. *prolixus*, we have identified what could be a critical problem for this biological tool: the parasite *T*. *cruzi* appears to confer a measure of resistance to the insect against the potential biopesticide agent so use of this fungus as a biopesticide could lead to selection for vector competence.

## Author Summary

Entomopathogenic fungi have potential for use in biopesticides for control of vectors of medical importance. As they are biological agents, however, a range of factors may affect their efficacy for this purpose, including temperature and interactions with the vectored parasite. Little is known about how the infection of entomopathogenic fungi affect the interaction between vectors of Chagas disease and the protozoan *Trypanosoma cruzi* but in other systems it is known that the efficacy of fungal pathogens can be modulated by the presence of microorganisms within the insect target. Here we screened fungal isolates as candidates for triatomine control, using as a model *Rhodnius prolixus*, vector of Chagas disease. We included in our screening assays of the virulences of these fungi over a range of temperatures and in insects infected with *T*. *cruzi*. Temperature affected the virulence of the fungi, indicating that some strains may be better thermal generalists than others (and so more versatile as biocontrol agents). Concentrating now on a single fungal candidate (a strain of *Beauveria bassiana*, we found that insects already infected with *T*. *cruzi* resisted the fungus for longer than did insects uninfected with the parasite over most of the temperature range tested. These results indicate a potential problem for the use of these fungi as biocontrol tools, as their use could generate selection pressure on the insect for greater chances of acquiring *T*. *cruzi*.

## Introduction

Insects of the subfamily Triatominae (Hemiptera: Reduviidae) are vectors of the protozoan parasite *Trypanosoma cruzi*, that is the causal agent of Chagas disease in Central and South America. This disease has a considerable medical and socioeconomic impact [1] with an estimated 7 to 8 million people affected by *T*. *cruzi* and approximately 12,000 deaths per year in the world [2,3]. The life cycle of *T*. *cruzi* is complex; the infection of mammals occurs when they come into contact with the infective metacyclic forms of the parasite that are eliminated with the feces of triatomines after feeding [4].

Interventions to manage triatomine vectors are based on their control using insecticides, particularly pyrethroids. However, insecticide resistance has been detected in some parts of South America, associated with ineffective treatments of deltamethrin against one of the most important vectors *Triatoma infestans* [5]. Isolates of entomopathogenic fungi have been reported to be highly infective to triatomines and potentially represent an alternative tool for controlling of Chagas disease vectors. These fungi can be highly infective to different insect life stages under laboratory conditions [6–9].

Selection of entomopathogenic fungi for use against insect pests requires consideration of factors such as the pathogen’s specificity, dose, host biology and environmental factors [10]. A highly virulent pathogen may require fewer propagules to cause the disease. However, fungal isolates with good persistence are more likely to come into contact with the target insect and so infect it [11]. The performance of fungi as biocontrol agents depends on environmental conditions as well as the behavioral responses of the insect targeted [12,13]. In this context, temperature could affect various biological parameters of triatomines [14,15] and also the development of the fungus inside the host [13]. Thus, it is of interest, in developing a fungal biocontrol agent, to have an understanding of how temperature affects the disease process.

A final consideration, and one of crucial importance for arthropod vectors, is that the aim of biocontrol is not primarily to exterminate or even control the insect populations but to manage transmission of the disease to the human host, for example by blocking parasite transmission or reducing vector longevity [16–18].

With this in mind, the first objective of the current study is to test the pathogenicity and virulence of isolates of entomopathogenic fungi against a triatomine vector of Chagas disease. For this, we assessed ten isolates of *Metarhizium* spp. and *Beauveria bassiana* against *Rhodnius prolixus* and then assessed the virulence of two of these isolates in a dose-response experiment. Our second objective was to examine isolate virulence across a range of temperatures, as a next step in our screening of isolates. Our third objective was to investigate the survival of insects infected by fungus when previously infected with *Trypanosoma cruzi*.

## Material and Methods

### Ethics statement

All experiments using live animals were performed in accordance to FIOCRUZ guidelines on animal experimentation and were approved by the Committee of Ethics of Animal Use-FIOCRUZ (L-058/08).

### Insects

*Rhodnius prolixus* were obtained from a colony maintained in the Vector Behaviour and Pathogen Interaction Group at the Centro de Pesquisas René Rachou (CPqRR), FIOCRUZ, MG, Brazil. *Rhodnius prolixus* were reared under controlled conditions of temperature (26±1°C) and relative humidity (65±10%). Insects were exposed to a natural cycle of illumination and allowed to feed weekly on chicken or mice.

### Production of spores and preparation of inoculum

We used 10 isolates of *Metarhizium* spp. and *Beauveria bassiana* (Bals.) (see S1 Table). Seven of these (C66A, J54A, J60A, L60A, C76B, S71B, L46C) were obtained from soil samples from coffee crops in Viçosa, Minas Gerais, Brazil, using live baits of mealworm beetles *Tenebrio molitor*, a standard procedure in our laboratory to isolate insect-pathogenic fungi [19]. The isolates C66A, J54A, J60A and L60A were identified as *Metarhizium robertsii*. Two isolates were kindly provided by the Federal Rural University of Pernambuco-Brazil, URPE-11 isolated from *Mahanarva posticata* (Hemiptera: Cercopidae) and URPE-18 obtained from soil. Isolate ENT-1 was from an unidentified coleopteran host from the Mata de Paraíso, Viçosa, Minas Gerais. The isolate URPE-11 was identified molecularly through the *Metarhizium* barcode region, the final portion of the 5’ translation elongation factor, using the primers EF1T and EF2T [20]. The isolate was identified as *Metarhizium anisopliae* by sequence blast search at NCBI (http://blast.ncbi.nlm.nih.gov/Blast.cgi) and alignment with other *Metarhizium* species at MEGA 6.06 [21].

All fungi were reactivated through inoculation of larvae of *T*. *molitor* and reisolated from the mycosed insect on Petri dishes containing PDA medium (20% Potato, 2% Dextrose and 1.5% Agar) and incubated at 25±1°C. Suspensions in sterile distilled water containing 0.01% of Tween 20 were prepared for each isolate and inoculated on rice. The rice had been autoclaved for 15 min at 120°C in polypropylene bags. Once cooled, a 2 ml aliquot of conidial suspension was added to 200g of the rice. The bags were maintained in an incubator at 25±1°C for ten days to promote sporulation. Rice with spores was placed in Falcon tubes with sterile distilled water containing 0.01% Tween 20. Suspensions were stirred for 3 min and filtered through sterile gauze so that the spores could be released. The suspensions were adjusted to standardized concentrations using a Neubauer hemocytometer, and were used immediately.

Conidial viability was assessing by adding 100μl of the suspensions on to Petri dishes containing PDA medium at 25±1°C. Viability was assessed after 20h by checking the germination of 100 conidia under an optical microscope (at 400x magnification). Conidial germination was over 85% in all cases.

### Experiment 1: Screening of ten fungal isolates for pathogenicity

We used first instar nymphs of *R*. *prolixus* of 2–3 days age for each fungal treatment. First instar nymphs were used in this experiment as they are expected to be more susceptible to fungal infection. As we are screening for pathogenicity, first instar nymphs can give a more rapid response for fungal pathogenicity to the insects. The nymphs were placed in Petri dishes (60x15mm) lined with filter paper containing 0.2 ml of fungal suspension (1x10^8^ spores/ml). Petri dishes were sealed with plastic film to maintain humidity and were maintained at 25±2°C, relative humidity of 80±5% and 12 h photophase. 48 h after exposure, the insects were transferred to new Petri dishes with untreated but humid filter paper. Insects were kept unfed in the Petri dishes until death. Dead insects were surface-sterilized (dipped in 70% ethanol then 2 min. in sodium hypochlorite then three washes in sterile distilled water) and were then placed in humidity chambers (within an incubator at 25±1°C) to promote fungal development and sporulation; hence confirming death by fungal infection. This procedure was done on the second day postmortem under a stereomicroscope (40x magnification).

This experiment was done in a randomized block design, with 12 treatments (ten isolates and two controls) and eight replicates per treatment. Each replicate had five insects. Controls were only water plus 0.01% Tween 20 (Control1) to check for cross-contamination or untreated filter paper to check for insect infections prior the experimental set-up (Control2). The methods used here for fungal inoculation and surface sterilization were also used in experiments 2, 3 and 4.

### Experiment 2: Concentration-mortality virulence bioassays of two fungal isolates

We used second instar *R*. *prolixus* nymphs, within 3–4 days post-moult, and exposed them to five conidial concentrations: 1x10^3^, 2x10^4^, 4x10^5^, 8x10^6^, 1.6x10^8^ spores/ml. Two isolates URPE-11 and ENT-1 were chosen based on their infectivity to *R*. *prolixus* (see results of Experiment 1 below) and high levels of sporulation on rice at 10 days (See S1 Appendix). The dilution series of both isolates were prepared in sterile distilled water with 0.01% Tween 20 and conidia were counted with a Neubauer hemocytometer. This experiment was conducted in a randomized block design with 12 treatments and six replicates per treatment with five insects in each replicate kept together in a Petri dish.

### Experiment 3: Effect of temperature on virulence of two fungal isolates

We used second instar *R*. *prolixus* nymphs at 3–4 days post-moult under four temperature regimes. We continued with the two isolates, URPE-11 and ENT-1, used in Experiment 2, at concentrations 1x10^3^ and 2x10^4^ spores/ml, respectively. These concentrations were selected as being borderline concentrations (based upon the results of Experiment 2 below) to allow the effects of temperature to be observed. Nymphs were kept unfed during the assays; inoculation was as in the previous experiments. The experimental design was as follow: insects were inoculated with one of the two fungal isolates or blank control (water plus 0.01% Tween 20) and kept at 21, 23, 27 or 30°C until death. Each experimental set-up was replicated six times and five nymphs were used for each replicate kept together in a Petri dish.

### Experiment 4: Effect of *Trypanosoma cruzi* infection on virulence of a single fungal isolate

Here, the parasite *Trypanosoma cruzi* (CL strain, isolated from a triatomine bug *Triatoma infestans*) [19], was used to test its effect on the virulence of a single fungal isolate. For this, second instar nymphs of *R*. *prolixus*, within 3–4 days post-moult, were fed on inactivated rabbit blood (37°C/20min) with culture epimastigotes (parasite) added at a concentration of 1x10^7^ parasites/ml. The control was only inactivated blood added with the same amount of culture medium used in the blood with contained parasites [22]. Upon feeding, insects were housed in a temperature-control chamber at 26°C until moulting to the third instar.

*Rhodnius prolixus* infections were conducted as routinely performed in one of our laboratories. Parasites are passaged through triatomines and mice every six months to maintain the strain infectivity [23]. Briefly, nymphs are infected with culture epimastigotes through artificial feeding. One month after infection, these insects are fed and their urine, containing metacyclic trypomastigotes, is collected and inoculated into a Swiss mouse. Two weeks after inoculation parasites are recovered by cardiac puncture and used to perform a hemoculture. As this procedure ensures insect infection rates from 85 to 100% (See S2 Table), we relied on this experience to assure infection rather than using direct tests—the normal procedure for confirmation of infection by *T*. *cruzi* requires that they be squeezed, which is sufficiently traumatic that it would have invalidated our experiments.

We used the third instar *R*. *prolixus* nymphs for the experiment, within 4–5 days post-moult and these were either parasitized or unparasitized by *T*. *cruzi*. *Beauveria bassiana* ENT-1 was used at a concentration of 2x10^4^ spores/ml. This isolate was selected due to its maintenance of virulence over a broader range of temperatures than the *M*. *anisopliae* isolate (see results of Experiment 3 below). The inoculation was as above. We used a factorial experimental design 2 x 2 x 4, as follow: insects (n = 4) were parasitized by *T*. *cruzi* or not and inoculated with a fungus (above) or not (control: sterile distilled water plus 0.01% Tween 20), and kept at 21, 23, 27 and 30°C until death. Each experimental set-up was replicated six times giving a total of 24 insects for each combination. Insects were kept unfed throughout the experiment.

### Statistical procedures

In all experiments, survival regression analyses were conducted in R software version 3.0.1 [24]. Data were analyzed by GLM with censored data with a Weibull distribution. Models were performed including fungal isolate, concentration, temperature and infection status (parasitized or unparasitized with *T*. *cruzi*) as independent variables. The function ‘frailty’ was used in the model to control the random effects, adding by blocks using Gamma distribution [25]. The models were carried out for all analyses and after exclusion of non-significant variables; the final model was accepted as the simplest model that was not significantly different from the full model. The mean survival time (lethal time = LT_50_) was calculated for each group for comparison between models by one-way analysis of variance (ANOVA) and the significance was observed using χ^2^ tests. Means were considered to be statistically different at *P* < 0.05. All insects, whether or not they sporulated after death, were included in the survival analysis, since it is common for fungi to infect and kill an insect, but not sporulate successfully from the cadavers [12]. A similar example is the entomopathogenic bacterium *Bacillus thuringiensis* which is well-known to kill insects but which is extremely difficult to recover in any number from cadavers [26].

## Results

### Experiment 1: Screening of ten fungal isolates for pathogenicity

**Fig 1:**
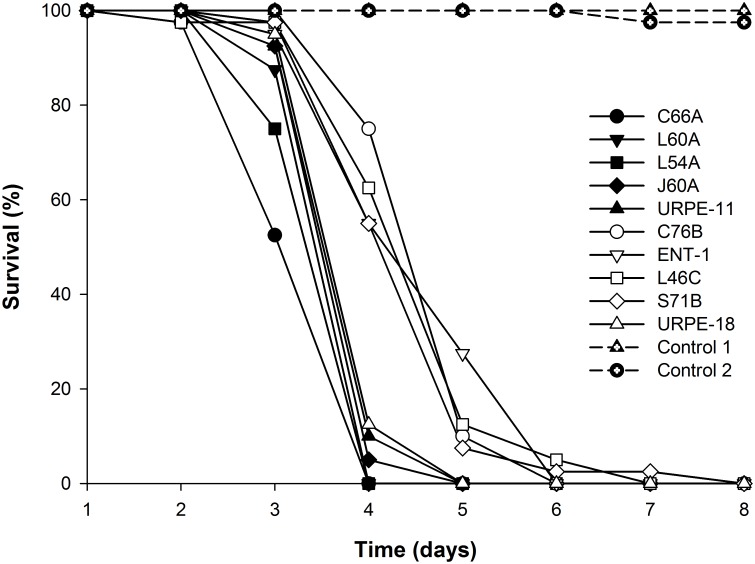
Screening of ten fungal isolates for pathogenicity. Survival of first instar nymphs of *R*. *prolixus* infected with the fungi *Metarhizium* spp. (isolates C66A, L54A, J60A, L60A, URPE-11) and *Beauveria bassiana* (isolates C76B, ENT-1, L46C, S71B, URPE-18). Control 1 is water+Tween 20; Control 2 is untreated filter paper.

All *R*. *prolixus* nymphs died eight days post-inoculation with the fungal isolates (Fig 1); LT_50_ varied from 3.7 to 5.3 days. Insects in the two control groups survived for the same length of time as one another (χ^2^_[01]_ = 2.804, *P* = 0.09) but considerably longer than the infected insects (7.98±0.09, χ^2^_[10]_ = 589.6, *P*<0.0001); only 5% of control insects had died by the 17th day post infection.

Comparisons among models showed that there were three groupings of isolates for which nymph survival times were statistically indistinguishable (*P*>0.05). The group that killed insects most quickly (LT_50_ 3.85±0.02 days) comprised *Metarhizium* sp. isolates C66A, L60A and L54A. The second group of isolates had an LT_50_ of 4.02±0.01 days, slower than the first group (χ^2^_[15]_ = 1096.1, *P*<0.0001), and comprised *Metarhizium* spp. isolates J60A, URPE-11 and *B*. *bassiana* isolate URPE-18. This group in turn killed insects faster than the third grouping, of *B*. *bassiana* isolates ENT-1, C76B, L46C and S71B (LT_50_ 4.79±0.01 days, χ^2^_[13]_ = 1019.4, *P*<0.0001).

Sporulation (as described above) was observed on 100% of dead insects for all *Metarhizium* spp. isolates and for *B*. *bassiana* isolates S71B, ENT-1 and C76B, whereas for *B*. *bassiana* isolates L46C and URPE-18, sporulation was 97.5% on dead insects. No sporulation was observed from control insects.

### Experiment 2: Concentration-mortality virulence bioassays of two fungal isolates

**Fig 2:**
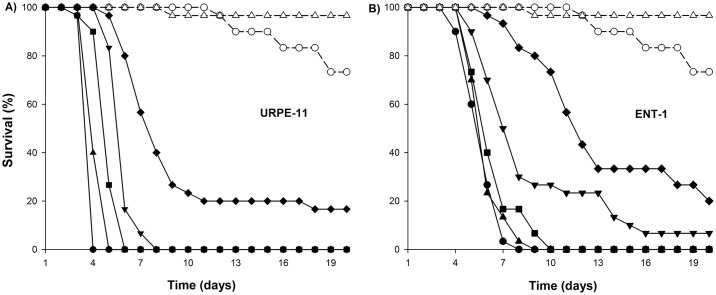
Concentration-mortality virulence bioassays of two fungal isolates. Survival of second instar nymphs of *R*. *prolixus* infected with different concentrations: 1.6x10^8^ (●); 8x10^6^ (▲); 4x10^5^(■); 2x10^4^ (▼) and 1x10^3^ (♦) spores/ml, plus Control 1 (○) and Control 2 (Δ) of **(A)** *Metarhizium anisopliae* URPE-11 and **(B)** *Beauveria bassiana* ENT-1.

Second instar *R*. *prolixus* nymphs were inoculated with five concentrations of *M*. *anisopliae* URPE-11 and *B*. *bassiana* ENT-1 and were monitored for 20 days. At all but the lowest doses, *M*. *anisopliae* isolate URPE-11 caused 100% mortality by the 7th day post infection (Fig 2A), with mean survival times of 3.96±0.23 (mean±SE), 4.4±0.23, 5.13±0.23, 6.06±0.23 and 9.96±0.24 days at concentrations of 1.6x10^8^, 8x10^6^, 4x10^5^, 2x10^4^ and 1x10^3^ spores/ml respectively. Survival curves of insects infected by fungi were significantly different from one another, these were different from controls (χ^2^_[10]_ = 476.0 for *P*<0.0001) and all were significantly shorter than untreated controls (χ^2^_[10]_ = 465.84, *P*<0.0001). With the exception of the two lowest doses, *B*. *bassiana* isolate ENT-1 caused 100% mortality by the 10th day (Fig 2B). Mean survival times were 5.8±0.23 (mean±SE), 6.1±0.23, 7.53±0.23, 10.06±0.23 and 13.46±0.23 days at concentrations of 1.6x10^8^, 8x10^6^, 4x10^5^, 2x10^4^ and 1x10^3^ spores/ml respectively. Survival times from the two higher doses were statistically indistinguishable from one another (χ^2^_[1]_ = 1.87, *P* = 0.17) while different from the three lowest doses and from controls (χ^2^_[09]_ = 385.57 for *P*<0.0001).

Sporulation was observed on 100% of dead insects at doses of 1.6x10^8^, 8x10^6^, 4x10^5^, 2x10^4^ spores/ml for the *M*. *anisopliae* treatment and on 84% of insects at the lowest dose (1x10^3^ spores/ml). For *B*. *bassiana*, 97% of sporulation on dead insects was observed at doses of 1.6x10^8^, 8x10^6^, 4x10^5^ spores/ml 79% for 2x10^4^ and 1x10^3^ spores/ml. No sporulation was found for any control insects.

### Experiment 3: Effect of temperature on virulence of two fungal isolates

**Fig 3:**
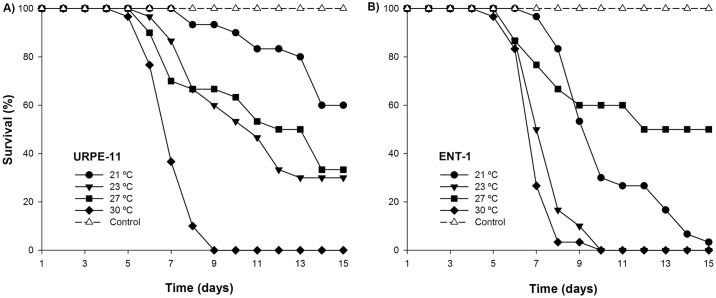
Effect of temperature on virulence of two fungal isolates. Survival of second instar nymphs of *R*. *prolixus* infected with **(A)** *Metarhizium anisopliae* URPE-11 and **(B)** *Beauveria bassiana* ENT-1 kept at four temperature regimes 21, 23, 27 and 30±°C.

For insects infected with *M*. *anisopliae* URPE-11, survival times generally decreased with increasing temperatures. At 30°C, mean survival time was 7.2±0.05 days. This was shorter than the survival times at 27 and 23°C (11.43±0.06 and 11.03±0.06 days respectively; χ^2^_[11]_ = 223.49 and χ^2^_[11]_ = 233.54 respectively, both *P*<0.0001) and mortality was 100% by the 9th day. There was no difference in survival of insects at 27 and 23°C (χ^2^_[1]_ = 1.153, *P* = 0.28). The survival times at 23 and 27°C were, in turn, shorter than survival at 21°C (13.83±0.08 days; χ^2^_[10]_ = 256.59, *P*<0.0001) with only 50% mortality of insects by the 15th days (Fig 3A). All of these survival times were significantly lower than controls (χ^2^_[10]_ = 251.85 *P*<0.0001).

For insects infected with *B*. *bassiana* ENT-1, survival times also generally decreased with increasing temperatures. At 30 and 23°C the mean survival times were statistically indistinguishable from one another (7.16±0.05 and 7.63±0.05 days, respectively; χ^2^_[1]_ = 1.47, *P* = 0.22) and mortality was 100% at 10 days (Fig 3B). These times were shorter than and statistically different from survival times at 21 and 27°C (10.4±0.03 and 11.6±0.06 days respectively; χ^2^_[10]_ = 353.49 and χ^2^_[09]_ = 175.56 respectively, both *P*<0.0001). At 21°C, survival time was shorter than that at 27°C (χ^2^_[10]_ = 374.56, *P*<0.0001). All of these survival times were significantly lower than controls (χ^2^_[10]_ = 359.12, *P*<0.0001).

Sporulation was observed on 100% of dead insects exposed at 21, 23 and 27°C for both isolates. At 30°C, sporulation was observed on 87% of dead insects infected with URPE-11 and 93% on infected with ENT-1 isolate. No sporulation was found for any control insects.

### Experiment 4: Effect of *Trypanosoma cruzi* infection on virulence of a single fungal isolate

**Fig 4:**
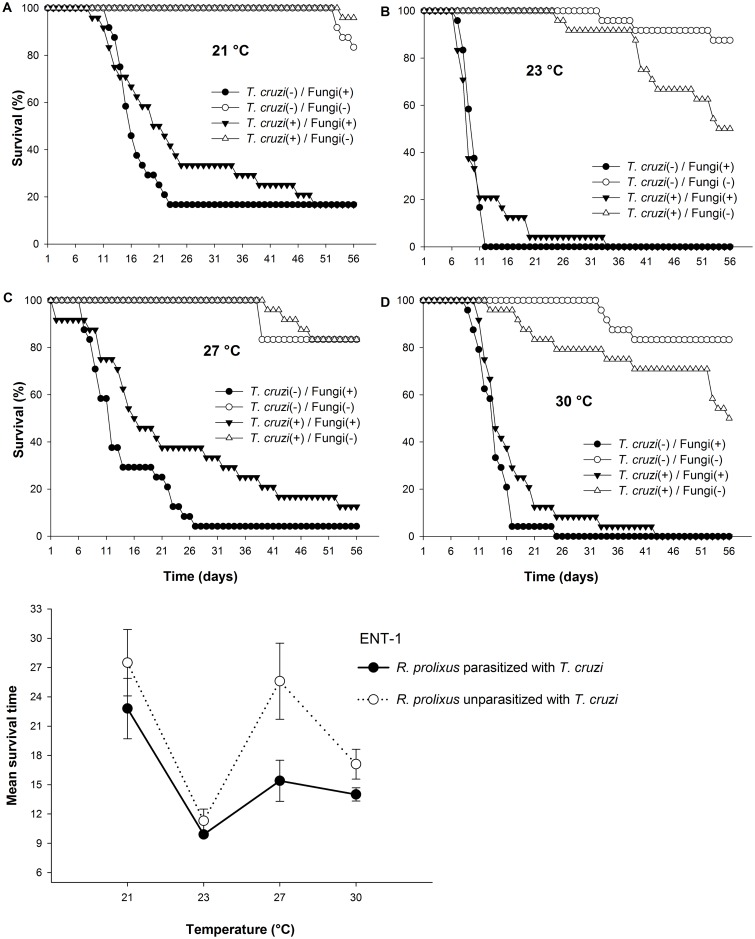
Effect of *Trypanosoma cruzi* infection on virulence of a single fungal isolate. Daily survival of *R*. *prolixus* infected with *Beauveria bassiana* ENT-1 (closed symbols) and *R*. *prolixus* uninfected control groups (open symbols) kept at four temperatures. Insects were either parasitized with *T*. *cruzi* (Δ▼ symbols) or not parasitized with *T*. *cruzi* (○● symbols).

*Trypanosoma cruzi* infection generally prolonged the survival times of insects when these were subsequently infected with the entomopathogenic fungus *B*. *bassiana* (ENT-1). The initial (full) model was simplified to keep only significant interactions. In the final model, there was no three-way interaction (fungal infection x parasite infection x temperature) (χ^2^_[3]_ = 4.136, *P =* 0.247). There were, however, two-way interactions between fungal infection and *T*. *cruzi* infection, between fungal infection and temperature and between *T*. *cruzi* infection and temperature (χ^2^_[16]_ = 511.24, *P<*0.0001) (Fig 4).

For the interaction between fungal infection and *T*. *cruzi* infection, the mean survival times of *T*. *cruzi*-parasitized versus unparasitized *R*. *prolixus* were lower when infected with *B*. *bassiana* (20.4±1.5 and 15.5±1.0 days respectively, χ^2^_[6]_ = 384.21, *P* <0.0001) than when not infected with the fungus (51.9±0.6 and 53.6±0.9 days, respectively, χ^2^_[6]_ = 5.34, *P* = 0.304).

For the fungal infection x temperature interaction, the lower mean survival times were recorded for insects infected with *B*. *bassiana* at 23 and 30°C, there was a significant difference between survival at these two temperatures (10.6±0.6 *versus* 15.5±0.8 days respectively; χ^2^_[10]_ = 455.4, *P*<0.0001). However there was no difference between survival times at 21 and 27°C (25.2±2.3 and 20.5±2.3 days respectively; χ^2^_[1]_ = 1.82, *P* = 0.18). For insects not infected with the fungus *B*. *bassiana*, no differences in survival times were observed at 23 and 30°C (52.2±1.0 and 49.5±1.7 days respectively; χ^2^_[1]_ = 1.61, *P* = 0.2), or between 21 and 27°C (55.7±0.1 and 53.6±0.8 days respectively; χ^2^_[1]_ = 1.06, *P* = 0.3). However, these two pairs were different from one another (χ^2^_[8]_ = 464.19, *P* = 0.02).

For the *T*. *cruzi* x Temperature interaction, there were two groupings of statistically equivalent mean survival times: (1) insects parasitized and unparasitized with *T*. *cruzi* at 23 and 30°C plus unparasitized at 27°C (χ^2^_[6]_ = 20.68, *P* = 0.25), and (2) insects parasitized and unparasitized with *T*. *cruzi* at 21°C plus parasitized at 27°C (χ^2^_[3.7]_ = 20.19, *P* = 0.54)

Sporulation was observed on 100% of dead insects parasitized with *T*. *cruzi* and unparasitized at all temperatures, except for insects parasitized with *T*. *cruzi* at 30°C for which sporulation was observed on 92% of dead insects. No sporulation was found for any control insects.

## Discussion

All fungal isolates tested here were pathogenic to *R*. *prolixus* nymphs, in line with previous studies [6,8]. *Beauveria bassiana* and *M*. *anisopliae* have a broad range of hosts, spanning numerous orders within Arthropoda [27] although individual isolates can be more restricted themselves differently according to the host.

The impact of the infection on nymph survival was fairly similar among groups of isolates, although *M*. *robertsii* group 1 (C66A, L54A and L60A) was more virulent than group 2: two *Metarhizium* spp. and one *B*. *bassiana* (URPE-11, J60A and URPE-18) and group 3 of *B*. *bassiana* isolates (L46C, C76B, S71B and ENT-1). Given infective isolates, the efficiency of mass production, feasibility of long-term storage and operational criteria are key in choosing an isolate for development [18]. Thus, URPE-11 (*M*. *anisopliae*) and ENT-1 (*B*. *bassiana*) were the candidate isolates selected for the dose and temperature bioassays as they required less time to sporulate on solid rice than the others (albeit in a qualitative observation), although they were not the most virulent of the isolates.

An important consideration in selecting a strain is its virulence, usually expressed as a median lethal dose or LD_50_ in the target insect [11]. Here, we found that *M*. *anisopliae* URPE-11 was the most virulent isolate against 2nd-instar nymphs, even at the lower concentration of 2x10^4^ spores/ml. This isolate negatively affected insect survival 4 days post-inoculation and caused 100% mortality by day 9. Meanwhile, 100% of insects inoculated with *B*. *bassiana* ENT-1 died at high concentrations by day 8. This comparison suggests that the *B*. *bassiana* isolate is less virulent to *R*. *prolixus* nymphs than *M*. *anisopliae*. It is important, however, to be aware that pathogens do not necessarily need to kill the insect rapidly, or even at all, in order to achieve the management goals [18], especially where these goals involve the control of a vectorborne disease.

It is important to consider how fungal biopesticides might perform when interacting with other variables in the field, such as variable temperatures and the presence of parasites (e.g. *T*. *cruzi*) in the host [13]. We found that the survival times of 2nd instar insects infected with *M*. *anisopliae* URPE-11 and *B*. *bassiana* ENT-1 decreased with the increasing in temperature. Those inoculated with *B*. *bassiana* ENT-1 isolate had 100% mortality at low and high temperatures. Meanwhile, the *M*. *anisopliae* isolate URPE-11, at first sight more virulent, caused less mortality at the lower temperature. It is likely that the origins of the isolates can explain the differences in virulence at varied temperatures. URPE-11 is originally from the northeast of Brazil, and may be better adapted to high temperatures, explaining its low virulence to insects at lower temperatures. Meanwhile ENT-1 was isolated in the southeast of Brazil where there is greater temperature fluctuation during the year, so the fungus may be a thermal generalist. These results may have some implications for control of Chagas disease vectors under field conditions. Given these considerations, it seems that ENT-1 isolate could be a better candidate for a biopesticide for management of Chagas disease vectors, although it would ultimately be of interest to examine daily fluctuating temperatures, insect behaviours and different insect species. According to Schilman and Lazzari [28] the mean preferred temperature for *R*. *prolixus* is 25.0°C for males and 25.4°C for females. When starved, these insects preferred slightly lower temperatures, approximately 24°C, and this preference only changed with prolonged food deprivation. The same pattern of thermopreference, i.e., a daily variation, as well as a decrease in preferred temperature with increasing starvation was also observed in two other triatomines *T*. *brasiliensis* [15] and *Panstrongylus megistus* [29]. So there are grounds to suspect that these factors may be important in determining the course of disease in these insect vectors. Overall, though, our initial conclusion is that *B*. *bassiana* isolate ENT-1 may be of more interest as a potential biopesticide.

For biopesticides as tools in vectorborne disease management, reduced longevity of the insect vector has been highlighted as a potentially useful consequence of infection [18], since it would prevent the transmission of parasites. An illustrative example is that *Aedes aegypti* and *Aedes albopictus* infected by a fungus, have their longevity reduced which limits viral development in the mosquitoes and thus reduces viral transmission [30]. Moreover, some studies show reductions in feeding of vectors such as mosquitoes infected with fungi [31–33], so it would be interesting to test if the bloodmeal affects the transmission of parasites.

Our final experiment was designed to examine this in the context of insects previously infected with *T*. *cruzi*. Contrary to our expectations, *T*. *cruzi-*parasitized insects survived longer than non-parasitized insects when exposed to *B*. *bassiana* ENT-1, independent of temperature. This is also in opposition to the background of a reduction in lifespan of *R*. *prolixus* caused by *T*. *cruzi* infection at 24 and 27°C, reported recently [23] and observed again in the *T*. *cruzi*-parasitized insects here at the higher temperatures—i.e. *T*. *cruzi* is virulent on its own but appears to reduce the virulence of a secondary, fungal, infection.

This type of effect of mixed infections has been observed before and depends entirely on which particular system is under study, which organism infects first, and a suite of other factors. A particularly relevant study is one in which the sand fly *Lutzomyia longipalpis* infected with the pathogenic bacterium *Serratia mercescens* was shown to survive longer when parasitized with *Leishmania mexicana* than without the parasite [34]. Those authors propose a mutualism between the insect vector and *L*. *mexicana* and indeed one might suppose that a parasite would protect its vector from secondary infections, even if it is virulent when as a single infection. While this promises to be an intriguing area of study, it is very early to form any firm conclusions.

It is known, however, that intestinal microbiota of vector insects can affect their competence as vectors and the process of parasite transmission [35–39]. Factors that have been implicated are digestive enzymes, hemolysins, agglutinins, microbiota and especially antimicrobial factors, which are potentially involved in regulating the development of *T*. *cruzi* in the gut [40]. As *T*. *cruzi* infects the triatomine gut, we may well expect it to affect infection by a fungal pathogen in another part of the insect: this effect of gut flora on fungal infection and disease has been known for some time, originally from locusts [41].

To conclude, we have found two fungal isolates of potential interest for development as biopesticides against *R*. *prolixus*. We have highlighted above some key areas of further study but the most important seems to be the three-way interaction between the insect, the fungal pathogen and *T*. *cruzi*, as our results so far indicate what could be a critical problem: if the parasite *T*. *cruzi* confers a measure of resistance to the insect against the biopesticide then the use of the biopesticide could feasibly lead to selection for increased vector competence. This would be a highly undesirable consequence of its development and use. This, then, is a key area for further study.

### Accession number

The sequence of fungal isolate *Metarhizium anisopliae* (URPE-11) is available at GenBank: accession number for the corresponding isolate is KX096871.

## Supporting Information

S1 Table Origins of fungi isolates. (PDF)

S2 Table Rate of infection of *Rhodnius prolixus* by *Trypanosoma cruzi*. *Rhodnius prolixus* nymphs infected routinely by Vector Behaviour and Pathogen Interaction Group at the Centro de Pesquisas René Rachou (CPqRR), FIOCRUZ, MG, Brazil.
(PDF)

S1 Appendix Isolates cultivated on rice for sporulation. **A)** *Beauveria bassiana* and **B)** *Metarhizium* spp. Photographs were taken six days after inoculation with *Beauveria* and seven days after inoculation with *Metarhizium*.
(PDF)
