# Supplementary material for: Screening of Fungi for Biological Control of a Triatomine Vector of Chagas Disease: Temperature and Trypanosome Infection as Factors
Source: PLoS Negl Trop Dis. 2016 Nov 17;10(11):e0005128. doi: 10.1371/journal.pntd.0005128 (PMC5113868; doi:10.1371/journal.pntd.0005128)
Supplement: S1 Table — (PDF) [file pntd.0005128.s001.pdf]

S1 Table. Origins of fungi isolates

Isolates	Specie	Host/substrate	Geographic/origin
**L54A**	*Metarhizium robertsii*	Soil/*Tenebrio molitor*	Minas Gerais/Brazil
**L60A**	*Metarhizium robertsii*	Soil/*Tenebrio molitor*	Minas Gerais/Brazil
**C66A**	*Metarhizium robertsii*	Soil/*Tenebrio molitor*	Minas Gerais/Brazil
**J60A**	*Metarhizium robertsii*	Soil/*Tenebrio molitor*	Minas Gerais/Brazil
**URPE-11**	*Metarhizium anisopliae*	Hemiptera: *Mahanarva posticata*	Recife/Pernambuco/Brazil
**C76B**	*Beauveria bassiana*	Soil/*Tenebrio molitor*	Minas Gerais/Brazil
**S71B**	*Beauveria bassiana*	Soil/*Tenebrio molitor*	Minas Gerais/Brazil
**L46C**	*Beauveria bassiana*	Soil/*Tenebrio molitor*	Minas Gerais/Brazil
**ENT-1**	*Beauveria bassiana*	Coleoptera	Minas Gerais/Brazil
**URPE-18**	*Beauveria bassiana*	Soil	Cabo/Pernambuco/Brazil
