# Supplementary material for: Screening of Fungi for Biological Control of a Triatomine Vector of Chagas Disease: Temperature and Trypanosome Infection as Factors
Source: PLoS Negl Trop Dis. 2016 Nov 17;10(11):e0005128. doi: 10.1371/journal.pntd.0005128 (PMC5113868; doi:10.1371/journal.pntd.0005128)
Supplement: S2 Table — Rhodnius prolixus nymphs infected routinely by Vector Behaviour and Pathogen Interaction Group at the Centro de Pesquisas René Rachou (CPqRR), FIOCRUZ, MG, Brazil. (PDF) [file pntd.0005128.s002.pdf]

S2 Table. Rate of infection of *Rhodnius prolixus* by *Trypanosoma cruzi*

Date	Insect infection (Positive/Total)	Percentage infection
19/05/2008	108/109	99%
29/07/2008	67/67	100%
12/02/2009	51/60	85%
5/10/2016	25/25	100%

Rhodnius prolixus nymphs infected routinely by Vector Behaviour and Pathogen Interaction Group at the Centro de Pesquisas René Rachou (CPqRR), FIOCRUZ, MG, Brazil.
