# Supplementary material for: Screening of Fungi for Biological Control of a Triatomine Vector of Chagas Disease: Temperature and Trypanosome Infection as Factors
Source: PLoS Negl Trop Dis. 2016 Nov 17;10(11):e0005128. doi: 10.1371/journal.pntd.0005128 (PMC5113868; doi:10.1371/journal.pntd.0005128)

S1 Appendix

Isolates cultivated on rice for sporulation: A) *Beauveria bassiana* and **B)** *Metarhizium* spp. Photographs were taken six days after inoculation with *Beauveria* and seven days after inoculation with *Metarhizium.*

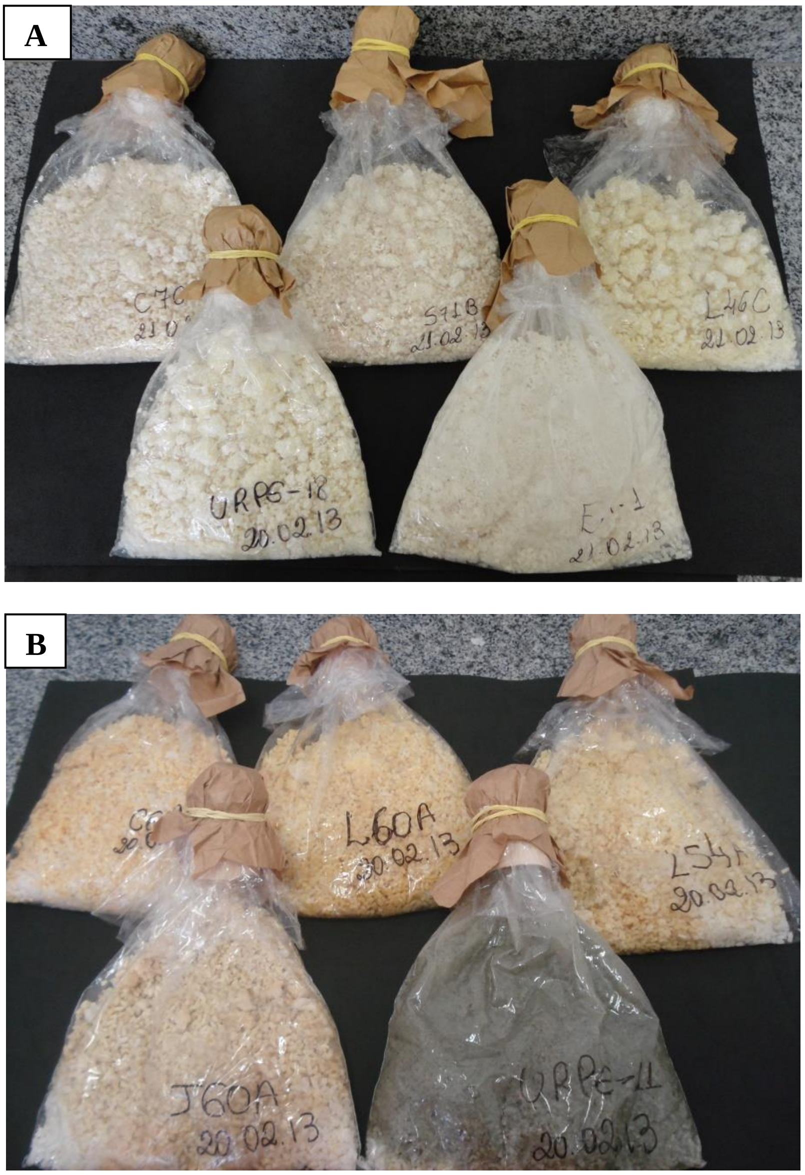

Supplement: S1 Appendix — A) Beauveria bassiana and B) Metarhizium spp. Photographs were taken six days after inoculation with Beauveria and seven days after inoculation with Metarhizium. (PDF) [file pntd.0005128.s003.pdf]
